# Internet-Based Interventions Aimed at Supporting Family Caregivers of People With Dementia: Systematic Review

**DOI:** 10.2196/jmir.9548

**Published:** 2018-06-12

**Authors:** Jenny Hopwood, Nina Walker, Lorraine McDonagh, Greta Rait, Kate Walters, Stephen Iliffe, Jamie Ross, Nathan Davies

**Affiliations:** ^1^ Centre for Ageing Population Studies Research Department of Primary Care and Population Health University College London London United Kingdom; ^2^ Research Department of Primary Care and Population Health University College London London United Kingdom; ^3^ eHealth Unit Research Department of Primary Care and Population Health University College London London United Kingdom; ^4^ Centre for Dementia Palliative Care Research Marie Curie Palliative Care Research Department University College London London United Kingdom

**Keywords:** dementia, caregivers, internet, review

## Abstract

**Background:**

Caring for someone with dementia is one of the most challenging caring roles. The need for support for family caregivers has been recognized for some time but is often still lacking. With an aging population, demand on health and social care services is growing, and the population is increasingly looking to the internet for information and support.

**Objective:**

In this review, we aimed to (1) identify the key components of existing internet-based interventions designed to support family caregivers of people with dementia, (2) develop an understanding of which components are most valued by caregivers, and (3) consider the evidence of effectiveness of internet-based interventions designed to support family caregivers of people with dementia.

**Methods:**

We conducted a systematic search of online databases in April 2018. We searched reference lists and tracked citations. All study designs were included. We adopted a narrative synthesis approach with thematic analysis and tabulation as tools.

**Results:**

We identified 2325 studies, of which we included 40. The interventions varied in the number and types of components, duration and dose, and outcomes used to measure effectiveness. The interventions focused on (1) contact with health or social care providers, (2) peer interaction, (3) provision of information, (4) decision support, and (5) psychological support. The overall quality of the studies was low, making interpretation and generalizability of the effectiveness findings difficult. However, most studies suggested that interventions may be beneficial to family caregiver well-being, including positive impacts on depression, anxiety, and burden. Particular benefit came from psychological support provided online, where several small randomized controlled trials suggested improvements in caregiver mental health. Provision of information online was most beneficial when tailored specifically for the individual and used as part of a multicomponent intervention. Peer support provided in online groups was appreciated by most participants and showed positive effects on stress. Finally, online contact with a professional was appreciated by caregivers, who valued easy access to personalized practical advice and emotional support, leading to a reduction in burden and strain.

**Conclusions:**

Although mixed, the results indicate a positive response for the use of internet-based interventions by caregivers. More high-quality studies are required to identify the effectiveness of internet interventions aimed at supporting family caregivers, with particular focus on meeting the needs of caregivers during the different stages of dementia.

## Introduction

Caring for someone with dementia can have a significant impact on the well-being of the caregiver. It is perceived as one of the most stressful and difficult forms of caring, as caregivers can face many years of managing difficult symptoms and making complex decisions [1,2]. Studies report higher levels of depression, emotional distress, and physical strain in caregivers of people with dementia than in caregivers for older adults with physical impairments [1,3].

There are around 670,000 family members and friends providing most care for people with dementia in the United Kingdom. Together, these caregivers are estimated to provide 1.3 billion hours of care per year and save the UK economy £12 billion annually[4]. Without the help of such caregivers, the formal care system would be likely to collapse [5].

With the given emotional and physical impact on caregiver well-being, psychological and practical support for caregivers is essential. There have been several trials of face-to-face interventions to support informal caregivers of people with dementia [6]. Reviews of interventions that provide information and advice have found varied results [7,8], but evidence of benefit has been found for some face-to-face psychological interventions in alleviating caregiver symptoms of depression [9]. However, uptake of such interventions is poor. It is estimated that around 10% of informal caregivers access caregiver support services [10], with the difficulty of leaving the care recipient and stigma being important barriers to uptake [11,12]. Individualizing caregiver interventions is also difficult economically, especially given the financial constraints in health care and the growing demand nationally and internationally due to the aging population [9].

Use of internet-based interventions may be an option to close the support gap for informal caregivers, particularly for those finding it difficult to leave their home or requiring flexibility due to caring responsibilities. Internet-based support interventions have the benefit of being relatively low cost and, by bringing the intervention into the home, may also have a role in reducing the social isolation that can come with caring [13,14]. Previous systematic reviews have suggested that internet-based interventions for informal caregivers of people with dementia have the capacity to improve various aspects of caregiver well-being, including depression, burden, and stress [15-17]. For psychological interventions in general, it is suggested that those with multiple components are better suited to support caregivers of people with dementia [9]. However, no previous reviews have identified what components might be important for interventions delivered via the internet for this group. Previous reviews have also focused predominantly on quantitative effectiveness data, which have been lacking in quality, and a mixed-methods review is important to provide richer data on how caregivers use and find benefit from internet-based interventions.

This review aimed to (1) identify the key components of existing internet-based interventions designed to support family caregivers of people with dementia, (2) develop an understanding of which components are most valued by caregivers, and (3) consider the evidence of effectiveness of internet-based interventions designed to support family caregivers of people with dementia.

Technology and digital health interventions is a fast-paced research field, and therefore previous reviews are now outdated and require updating. Previous reviews have also focused on the effectiveness of whole interventions, where there are limited data to draw such strong conclusions, and in doing so have neglected a thorough and clear description of the content of interventions and their acceptability by caregivers.

## Methods

### Design

We conducted a systematic review of randomized controlled trials (RCTs), quasi-experimental designs (pre-post studies), quantitative studies, and qualitative studies, following the guidelines from the Centre for Reviews and Dissemination [18].

### Inclusion and Exclusion Criteria

We included articles if they met the following criteria: (1) the intervention was aimed at informal caregivers (defined as a family member or friend providing unpaid care) of people with dementia, (2) the intervention was a digital intervention delivered via the internet, and (3) the article considered a specific intervention and provided a description of this.

We excluded articles if (1) the intervention was telephone or telehealth based, (2) the interventions solely used Skype or another means of online calling, (3) the intervention had a large face-to-face component, (4) results or outcomes of the intervention were not reported, (5) the intervention was focused on the person with dementia, or (6) the study was not published in a peer-reviewed journal.

As our interest was in digital technologies that could be used by caregivers without input from health professionals, we excluded telephone-based support and those interventions with a large face-to-face component.

### Search Strategy

We conducted a systematic literature search in CINAHL, the Cochrane Library, EMBASE, MEDLINE, PsycINFO, and Web of Science for articles published between January 1990 and April 2018. We selected 1990, as this was the period when the internet, including email, started to develop in commercial and public settings.

**Figure 1:**
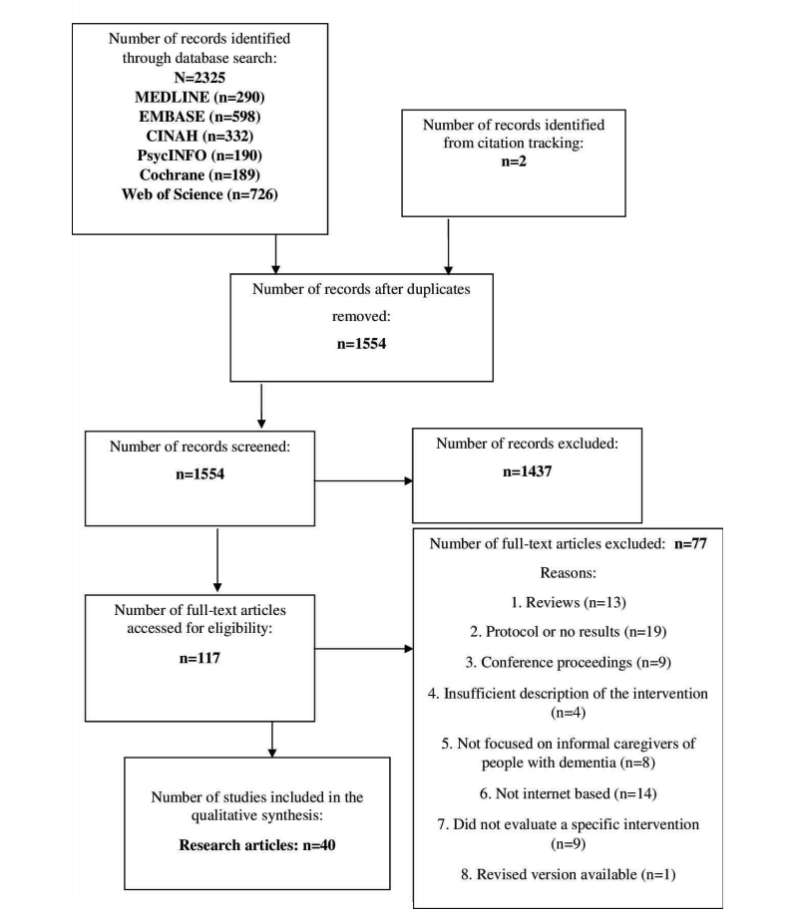
Preferred Reporting Items for Systematic Reviews and Meta-Analyses (PRISMA) flowchart describing the search process for articles on digital interventions for caregivers of people with dementia.

We tracked citations using Google Scholar, and hand searched reference lists for any additional relevant articles, in addition to hand searches of relevant journals. We identified literature reviews on the topic and checked them to ensure that our search identified relevant articles. Search terms and index terms (Medical Subject Headings) were identified from the initial scope of the literature. We added synonyms or abbreviations that we felt were appropriate to the search terms. Multimedia Appendix 1 shows an example search strategy from MEDLINE. We contacted experts in the field. We also included gray literature, including reviews of websites, in the scoping work but not in the review.

### Selection Procedure

Article titles and abstracts were screened and excluded if they did not meet the inclusion criteria by 2 reviewers (JH and ND). We rapidly appraised non-English language articles, using their English abstracts, to ensure that we did not exclude any important articles. Articles considered relevant or where insufficient information was supplied in the abstract and title were read in full by 2 reviewers (JH and ND). Two reviewers enhanced the validity and reliability of the selection procedure [18]. Any disagreement between reviewers or uncertainty about inclusion of articles would have been decided by a third reviewer, although this was not required. Figure 1 shows a Preferred Reporting Items for Systematic Reviews and Meta-Analyses flowchart detailing the selection process.

### Quality Appraisal

We appraised the literature for quality using the Critical Appraisal Skills Programme tools, using different tools for varying study design, qualitative studies [19], and RCTs [20], and an adapted version of the Critical Appraisal Skills Programme toolkit for quantitative designs [21]. We excluded no studies based on the results of their quality appraisal; rather, we used the study appraisal to develop discussion of the included studies.

### Data Extraction and Synthesis

We developed a standardized data extraction tool to examine the included studies. We extracted data on study design, intervention details and components, duration of the intervention, participant characteristics, outcome measures, key findings, and the conclusions drawn by study authors. As the review included both quantitative and qualitative studies, and quantitative designs were heterogeneous, we could not pool quantitative data to conduct a meta-analysis. Therefore, we performed a narrative synthesis, using tabulation to organize the studies and a thematic analysis to categorize and group the studies. Two reviewers independently coded all studies (JH and ND). The 2 reviewers met to discuss each of their coding frames, discuss any disagreements, and develop a refined coding frame. Using the refined coding frame, all studies were coded by 1 reviewer (JH), and a selection of articles (50%) were blindly coded by a second reviewer (ND) and checked for agreement. Any disagreement was discussed and a third researcher would have been consulted if agreement could not be reached, although this was not required.

## Results

### Description of Studies

We included 40 articles [22-61] addressing 31 different interventions. There were 9 RCTs [22-30] (Multimedia Appendix 2), 7 quasi-experimental studies [31-37] (Multimedia Appendix 3), 4 qualitative studies [38-41] (Multimedia Appendix 4), and 20 studies with mixed or other methods [42-61] (Multimedia Appendix 5). All statistics in the multimedia appendices are reported as per the original articles.

All interventions aimed to address the needs of informal caregivers of people with dementia. One intervention also provided support for professional caregivers [26] and 2 provided support for people with dementia [41,49]. Some interventions limited their inclusion population to address specific caregiver needs, including 3 bilingual websites that addressed the needs of caregivers from minority ethnic groups [46,56,61]. Some restricted their intervention to informal caregivers experiencing stress [23,36,47], burden [24,29,57], depression [24,57], or anxiety [24,57].

Most interventions aimed to address the needs of caregivers providing care to people with all stages and types of dementia. One limited the intervention to spousal caregivers of people with mild cognitive impairment or “mild” dementia [44], 1 was limited to caregivers of people who were housebound with dementia [25], and 2 interventions also included people caring for those with other forms of neurodegenerative disease [36,39,40,52].

Although all interventions were primarily internet based, some had supplementary telephone-delivered components; for example, 1 provided a telephone number on their website for caregivers to contact a health care professional [32], and 1 included monthly telephone calls with caregivers [45].

A large number of different outcome measures were used in the studies. Outcomes included data on the usability of the interventions, as well as impacts on well-being, quality of life, burden, competence, physical health, and mental health. A wide range of validated and nonvalidated rating scales were used to assess the impact of the interventions. Qualitative results mainly focused on usability of the interventions and included observation of use [39-41,44,49], free-text surveys [38,41,49,51,55,61], interviews [38-41,46,47,47,53,54,59,60], and focus groups [42,45,56].

### Quality Appraisal

The articles were of variable quality. Sample sizes ranged from 5 to 299, with many studies being pilot or feasibility studies. A problem with possible selection, performance, detection, or attrition bias was identified in many of the studies; many studies had small convenience samples, high attrition rates, and poor descriptions of randomization in trials and of data collection; and in some cases data collection was completed by nonblinded members of the study team.

### Themes

We identified 5 themes as key components of the interventions: peer support; contact with a health or social care provider; provision of information; decision support; and psychological support.

#### Peer Support

Peer support was a key component of the interventions in 25 of the studies [22,25,26,29,31-33,36,38-42,44,45,47-50,52-56,58]. Peer support provided by fellow caregivers online was delivered either in private or in public, where all individuals using the intervention could see interactions. Common uses of peer support included supportive messages, information seeking, discussing the emotional impact of caring, and developing support networks outside of the Web-based intervention.

Private peer support was provided via private email or an online messenger service [22,36,39,40,45,48,49,52,53,55]. For example, the Digital Alzheimer Center allowed users to find others in their area caring for someone with the same diagnosis and then to communicate via private messaging [49]. However, in the few studies that quantified use of private messaging, use varied from very infrequent [49] to being one of the most-used functions [40].

One intervention, Inlife, provided the opportunity for the primary caregiver to develop their own networks of online support with friends, family, or significant others [48]. This allowed them to develop care books providing an overview of contact and practical information regarding the care of the individual, transfer care tasks among individuals, and provide help and assistance to one another.

Some interventions provided peer support in small groups [42]. For example, O’Connor and colleagues developed a virtual reality support group within an avatar environment [55] where groups of 3 to 4 anonymous caregivers communicated via online text. The groups were driven by the caregivers, allowing for exchange of ideas about communication, caring, and information about dementia, with some direction provided by a psychologist. Outcomes evaluated included loneliness, depression, burden, and perceived stress, but the study was underpowered to demonstrate any effects. A similar approach using videoconferencing software was used in another intervention where groups of caregivers met weekly online, initially with a facilitator, then as a peer group alone [39]. More than 90% of caregivers found this a positive experience, and there was a significant decline in stress in the experimental group. Use of the internet to deliver the intervention was felt to be as helpful as meeting people face-to-face by 61% of participants [52]. When this videoconference support group was compared with an internet-based chat group [53], both groups had a significant improvement in self-efficacy, but the video group showed a significantly greater improvement in mental health status. However, this was a pre-post study design with a duration of 6 months.

Public peer support usually consisted of forums [22,29,36,39,40, 44,45,47,49,50,52-54,56,58] but also included chat rooms [32,41], shared blogs [25], links to peer groups on social networking sites [26], and video messages [31]. However, use of these tools was variable. Some studies reported that forums were not well used and were negatively reviewed by participants in qualitative reports [29,44]. In 1 study, this was thought to be due to the forum having an unclear purpose, the anonymity of participants, and a perceived high threshold for starting conversations [44]. In another study, 76% of participants visited the forum fewer than 12 times over the 12-week study [54]. However, some studies reported positive views, good rates of use, and a good impact on caregiver outcomes. For example, a 12-month RCT from Bass and colleagues analyzed the impact of the communication function and demonstrated a reduction in physical and emotional strain associated with use of the communication functions for caregivers who were initially under the most strain [22]. McKechnie and colleagues found a statistically significant improvement in the quality of the relationship with the care recipient but found no impact on depression or anxiety [54]. However, this was a smaller pre-post study with a short intervention period of only 12 weeks. Qualitative data suggested that participants found many benefits from peer interaction, including feeling understood through shared experience, finding reward in helping others, having reduced isolation, and being able to access information that would be difficult to find elsewhere [39,42,45,52,54]. However, in the 1 study where social isolation was measured using a validated scale, peer interaction did not demonstrate a significant benefit [45].

Most interventions that provided private peer interaction also provided the option for public interaction [22,36,39, 45,49,52,53]. When comparing private peer interaction with public peer interaction, Brennan and colleagues found that the public forum was used with increased frequency and duration compared with the private mail function [45], a contrast to findings from the Digital Alzheimer Center [49]. In qualitative feedback, participants found it difficult to recall the email addresses of others when using this private mail function so preferred to interact publicly.

The studies suggest that functions that have the potential for visual contact or group interaction may be more promising than simple chat-based functions in improving mental health status.

#### Contact With Professionals

Of the studies, 11 included components to allow caregivers to have direct contact with and ask questions of either a health or a social care professional [22,25,32,41,45,46,49,56,58-60]. Professionals included nurses [22,45], occupational therapists [46], or social workers [27,46,58]. In some interventions, the role of the professional was not clear; rather, the caregiver was described as having contact with a “medical professional” or “expert” [32,41,49,56,59,60], or a multidisciplinary team [25].

Most interventions required caregivers to contact health professionals themselves [22,25,32,45,46,49,56,58-60]. For example, the eHealthMonitor dementia portal [59,60] provided alerts for health professionals when caregivers entered a question; professionals could then respond online or arrange an appointment via telephone. Only 1 intervention adopted a proactive approach where health professionals contacted caregivers who self-assessed as having severe stress [27]. The intervention as a whole led to a significant decrease in hardship and grief compared with the control group, but there was no significant change in burden, depressive symptoms, or desire for nursing home placement.

On the whole, evaluation data from the studies showed that interaction with professionals was a positive experience for caregivers [45,46,58-60]. Professionals provided personalized practical advice for caregivers at home on caring and dementia, as well as emotional support, and caregivers reported feeling less isolated as a result. However, opinions about seeking this support electronically did vary [46,56], with some caregivers enjoying writing emails, while others felt confused about how much information to include.

#### Provision of Information

Most interventions provided information for caregivers about dementia, practical aspects of caregiving, or available local and national services. For some, this was the only function of the intervention [30,37,51,61], but for most information provision was part of a multicomponent intervention [22-29,32-36,38-40, 42-50,52,53,56-60]. Some RCTs of multicomponent interventions that included the provision of information did demonstrate positive impacts on depression [24], anxiety [24], perceived stress [28,52], and attitudes toward dementia [26]. However, as information was part of a broader intervention, it was difficult to know the impact of this component. One intervention that was analyzed in an RCT that attempted to assess this was ComputerLink [22], which provided information on dementia, caregiving, and local services as part of a multicomponent intervention. Use of the information provision parts of the intervention was associated with reduced strain for caregivers living alone with care recipients and for spousal caregivers. However, other multiple-component interventions evaluated with qualitative methods found that caregivers found other components, such as interaction with professionals, more beneficial than information [40,46], with caregivers expressing frustration when required to review information that did not meet their specific needs [38,56]. When information was individualized, it was considered by caregivers as one of the most useful functionalities of the intervention [37,59]. This suggests that information does appear to be an important part of interventions, but the information should be tailored to the individual caregiver situation and not be the sole focus of the intervention.

#### Decision-Making Support

Some of the interventions recognized that decision making is a difficult process for caregivers and included decision aids [22,36,41,45,59,60]. However, most studies did not explain in detail how the intervention provided support with decision making; for example, Lorig and colleagues included decision-making assistance in their online workshops and chat forums [36] but lacked further description of how this was achieved. The only well-described decision aid intervention was ComputerLink [45], which included a tool based on multiattribute utility theory [62], where caregivers were led through a series of questions prioritizing important factors in the decision-making process. Use of the decision-making tool significantly improved caregiver confidence prior to having face-to-face discussions when compared with the control group. However, in some studies the decision-making tools were poorly used [41,45] and not appreciated by caregivers [41]. Instead, participants gained decision support from other components of the interventions, such as discussion with peers or professionals [41,45].

#### Psychological Support

Many interventions included components of psychological support [23,24,26-29,32-36,39,44,46,47,55], which were self-guided or professionally guided. Few used standardized forms of psychological interventions or therapy, but therapeutic relaxation techniques were commonly used.

Self-guided psychological support most often consisted of modules that caregivers worked through, and several were tested in RCTs. For example, Beauchamp and colleagues delivered a modular intervention that provided videos on cognitive and behavioral strategies to cope with difficult emotions [23]. In an RCT of the intervention, the experimental group had significantly greater improvements in stress, self-efficacy, intention to get support, strain, gain, depression, and anxiety. Similar results were found in other RCTs of similar psychological interventions, with reductions found in caregiver stress in 1 intervention [28] and improvements in attitudes toward dementia, distress, empathy, and perspective in another [26]. However, the durations of these RCTs were short, ranging from 1 to 4 months.

Some interventions provided self-directed modules to work through, but caregivers were supported by a professional coach, who was most often a psychologist [24,33,38,44,57]. Caregivers were required to complete assignments, homework, reflective diaries, or regular assessments of their well-being. An RCT of 1 such intervention showed a reduction in symptoms of anxiety and depression with moderate and small effect sizes, respectively [24].

Some studies provided professionally delivered psychological therapies online, either via individual interaction with a therapist using email [35,46] or online interaction with a small group of caregivers [32,36,39,40,52,53,55]. In the ADCarer.com intervention [35], the professional (a psychologist, social worker, or counsellor) would respond to online messages from the caregiver within 48 hours using cognitive behavioral therapy techniques. In a pre-post assessment, the multicomponent intervention did lead to a significant reduction in caregiver distress. Interactive groups were delivered either using videoconferencing software [32,39,40,52,53] or an avatar-based format [55] and allowed small groups of caregivers to interact, guided by a professional. Improvements were found in caregiver mental health and quality-of-life outcomes, but with these interventions as with many others, it is difficult to tease out the specifics of components, as in both cases the virtual support group offered peer support as well as psychological support.

Overall, studies assessing psychological support suggested a positive effect on a variety of factors, including improving caregiver distress, depression, anxiety, and strain. However, some stressed the importance of cultural appropriateness. Kajiyama and colleagues used the popularity of Spanish-language telenovela (a type of television serial drama or soap opera produced mainly in Latin America) to appeal to Hispanic and Latino family caregivers [34].

## Discussion

### Principal Findings

Unlike previous reviews in this area, this review explored the key components of internet-based interventions to support family caregivers of people with dementia. We identified a broad variety of interventions, which focused on providing peer support, engaging with health and social care professionals, and providing information, decision support, and psychological support. Although effectiveness was not a focus of this review, some multiple-component interventions showed promise in reducing stress, anxiety, and depressive symptoms for family caregivers and in increasing self-efficacy [44,57]. However, as with previous reviews [15-17], the limited number of high-quality RCTs, as well as the multiple-component nature of many interventions, makes it difficult to report which aspects of the interventions were effective.

Peer support was a key component of many of the interventions discussed. Caring for someone with dementia has often been described as not only a lonely role but also one in which there is a great deal of uncertainty. The peer support components of the interventions identified in this review aimed to target these feelings and were described positively by many participants, but no significant effect for peer interaction and social isolation was found [45]. However, qualitative data in this review suggest that peer support offered a form of socialization. Previous evidence is mixed on whether use of the internet reduces or enhances loneliness [63,64], but this review suggests that internet-based peer interaction may have a benefit for family caregivers. However, it is evident that the way that peer support is delivered is important, with opportunities for group interaction or videoconferencing being more beneficial than public-facing forums and private messaging functions.

The qualitative data suggest that interactions with health professionals are viewed positively; however, it is unclear whether this positivity was linked to the provision of contact online or whether caregivers may prefer this interaction face-to-face. The mix of professionals providing support in the studies suggests there is a lack of consensus on who is best to deliver professional support. This may reflect ambiguity caregivers feel about who is the most appropriate person to talk to when they need advice.

The provision of information was often at the core of interventions, and this supports findings from previous research where most caregivers preferred to receive information online rather than in paper format [65].

This review demonstrated that interventions that focused solely on decision making were, in general, not favored by family caregivers. However, decision-making tools were viewed more positively when they were used alongside other components, such as peer support. Decision making is often left to family caregivers when the person with dementia no longer has capacity, making this a difficult and challenging time for family caregivers. However, results from this review suggest that face-to-face meetings may be required to make decisions, and internet resources are only used as a method of preparation for discussions. This adds to our understanding of barriers to making decisions, which include a lack of information, poor communication, difficult dynamics and conflict within families, and limited emotional and practical support [66-70].

Interventions including online psychological support showed some of the most promising findings, with individual studies reporting significant reductions in caregiver stress, strain, depressive symptoms, and anxiety, in addition to increases in self-efficacy [23,26,28]. Although studies of both professionally guided and self-guided interventions indicated a positive outcome for participants, including caregiver mental health outcomes, they were quasi-experimental (pre-post studies), feasibility studies, and small RCTs, suggesting these conclusions should be made with caution.

Qualitative evaluations of the interventions demonstrated positive views from most caregivers toward internet-based support interventions, although it is clear that not all would benefit from such interventions. It may be that the internet is most beneficial for those who are classified as most vulnerable (ie, more stressed) [22].

### Implications for the Development of Future Internet-Based Interventions for Caregivers

In developing an internet intervention for family caregivers, several issues need to be addressed. Questions of privacy and security were highlighted [59,60], reflected in the contrast of public versus private messaging approaches and password-protected websites. The details discussed by many on the websites are very personal and emotional topics. Sillence and colleagues discussed a series of factors that influence the mistrust and trust of health websites [71]. The design of the site contributed to most of the reasons for rejecting and mistrusting a website, including complex and busy layout, corporate look, and irrelevant content. However, the reasons for selecting and trusting a website were more focused on the content of the website, including unbiased information and personalized content.

Another issue is complexity. Some caregivers found functions such as private messaging, decision aids, and login screens complex, which affected their use. Using familiar-sounding language [71] and a strong iterative approach, in which the intervention undergoes multiple cycles of development and optimization [72], with future interventions are two ways to help overcome this challenge Tailoring can reduce the quantity of information and resources caregivers must review, and caregivers may be more motivated to use an intervention they feel is applicable to their circumstances. This review found that where interventions were not personalized, caregivers found this frustrating and their needs were not met [47]. Finally, there is the question of internet literacy and access to the internet: the digital divide [73]. There appears to still be a gap between those who use or can use the internet and those who don’t, with a study in 2015 highlighting that almost all adults over 70 years of age had difficulty using the intervention [41]. Many of the studies included in this review consisted of participants who were predominantly younger caregivers, whereas many people caring for someone with dementia are more likely to be older. Reducing the complexity of interventions, supporting access with potential support from health professionals, and highlighting the benefits of such interventions to understand their potential value may aid in bridging the divide. For most of the studies, this digital divide was ignored, as a requirement for participation was computer literacy [61], and observational studies assessed the usability of the interventions with caregivers who had already received training in using the website.

### Implications for Policy, Clinical Practice, and Further Research

This review demonstrated the need for high-quality research to evaluate the effectiveness of internet-based interventions for caregivers of people with dementia, in particular larger phase 3 trials. Importantly within these studies, it would be useful to describe the interventions in more detail and to understand which aspects of the interventions are used more than others and provide the most benefit. Future research should also focus on which aspects of the interventions are most beneficial for different groups—for example, adult children compared with spouses—and how the interventions can best be delivered to address issues such as the digital divide. Future research would also benefit from including theoretical considerations of how interventions are thought to provide support to caregivers.

This review identified a gap in the development of interventions targeting specific stages of the dementia trajectory. Many of the interventions in this review were broad and generic to the entire dementia trajectory. However, the needs of family caregivers vary at different stages of the disease and transition points; for example, around the end of life of the person with dementia, caregivers face specific challenges around decision making and management of difficult symptoms. Future interventions and research should address these different stages when developing digital interventions to support family caregivers [49].

### Strengths and Limitations

Similarly to previous reviews in this area [15-17], comparison between studies was difficult, as the interventions used were complex and varied, with wide-ranging study designs and outcome measures. The review was also limited by the quality of some of the studies and the methods employed. There were relatively few RCTs from which to derive effectiveness data. Many of the studies were feasibility and pilot studies, so we were unable to draw definitive conclusions surrounding effectiveness and acceptability. For many of the studies, there were high levels of dropouts and for some interventions participants made limited use of some of the components of the interventions, therefore making it difficult to draw conclusions [46,49]. Few studies provided information on the effectiveness of individual components of the interventions, and some studies explored only usefulness and usability with reference to the design and layout of the interventions, which on the whole were not well described. This is helpful only to an extent because, to develop or build on existing interventions, there needs to be an understanding of which elements have a positive effect on family caregivers and so should be included in new interventions.

Our literature search was limited by including only peer-reviewed publications, and there may have been several other interventions that were being practically used and applied but not published via academic routes. However, the search of the academic literature was thorough and we used a rigorous search strategy, updated before publication.

This review has built on previous literature by identifying the core components of interventions for family caregivers, which will be useful for future intervention development. As our inclusion criteria were much more comprehensive, this review provides a larger evidence base than previous reviews. Unlike previous reviews, we have particularly considered how caregivers are supported with decision making through Web-based interventions and we included data from many qualitative studies, providing richer information on how the interventions were perceived and valued by caregivers.

### Conclusions

The evidence base for internet-based interventions for caregivers of people with dementia remains limited. Although this review recognizes that for some caregivers, a face-to-face intervention may be preferred, our findings highlight the promising potential of digital interventions to support caregivers, which warrants further development and testing.

## Appendix

Multimedia Appendix 1 MEDLINE search terms and strategy.

Multimedia Appendix 2 Characteristics, components, outcomes, and key findings of randomized controlled trials.

Multimedia Appendix 3 Characteristics, components, outcomes, and key findings of quasi-experimental (pre-post) studies.

Multimedia Appendix 4 Characteristics, components, outcomes, and key findings of qualitative studies.

Multimedia Appendix 5 Characteristics, components, outcomes, and key findings of mixed-methods and other methods studies.

## Abbreviations

RCT: randomized controlled trial
