# Supplementary material for: Internet-Based Interventions Aimed at Supporting Family Caregivers of People With Dementia: Systematic Review
Source: J Med Internet Res. 2018 Jun 12;20(6):e216. doi: 10.2196/jmir.9548 (PMC6019848; doi:10.2196/jmir.9548)
Supplement: Multimedia Appendix 1 [file jmir_v20i6e216_app1.pdf]

MEDLINE search terms and strategy

1. care giver*.tw.
2. caregiver*.tw.
3. carer*.tw.
4. ((family or families or spouse* or relative* or partner$1 or proxy) adj10 (caring or care* or support*)).tw.
5. or/1-4
6. *Caregivers/ or exp Family/ or Spouses/
7. or/5-6
8. (ehealth or e health or m health or mhealth or mobile health or ("electronic health" not "electronic health record") or internet).tw.
9. ((mobile or phone or smartphone or apple or android or computer or digital or online or on-line or electronic or tablet or ipad or "personal digital assistant" or internet) adj5 (app or apps or application$1 or program* or interface$1 or tool* or web*)).tw.
10. (telehealth or telemedicine).tw.
11. or/8-10
12. exp *Telecommunications/
13. exp Computers/
14. exp *Internet/
15. exp Mobile Applications/
16. exp therapy, computer-assisted/
17. or/11-16
18. (support* or information or advis* or advice or intervention* or therap* or program* or train* or educat*).tw.
19. exp *social support/
20. or/18-19
21. exp *Dementia/ or exp *Cognition Disorders/
22. (dementia* or alzheimer* or cognitive impairment or neurodegenerative).tw.
23. or/21-22
24. 7 and 17 and 20 and 23
25. limit 24 to (english language and yr="1990 -current")
