# Supplementary material for: Internet-Based Interventions Aimed at Supporting Family Caregivers of People With Dementia: Systematic Review
Source: J Med Internet Res. 2018 Jun 12;20(6):e216. doi: 10.2196/jmir.9548 (PMC6019848; doi:10.2196/jmir.9548)
Supplement: Multimedia Appendix 2 [file jmir_v20i6e216_app2.pdf]

First author, year, country, reference	Study design, duration	Population	Core components	Control group	Outcome measures	Key findings
Bass, 1998, US [22]	RCT<br>12 months	Primary informal caregivers of community-dwelling older people with Alzheimer disease (N=102); mean age 60 years	1. Peer interaction via a bulletin board and private mail<br>2. Online question and answer with a nurse<br>3. Information on AD, treatment, and caring<br>4. Decision support tool	1 face-to-face information session on caregiving and Alzheimer disease	Physical strain; emotional strain; relationship strain; activity restriction; frequency and duration of use	Peer and nurse interaction sections more often used than solitary parts (average 11.28 and 0.97 times per month, respectively).<br>Reduced relationship strain for spouses and emotional strain for those with more social support.<br>Duration of use of the interaction functions associated with reduced strain in those with most initial strain and non–spouse caregivers.
Beauchamp, 2005, US [23]	RCT<br>30 days	Employed family caregivers with regular caring duties who reported experiencing stress as a result of caring (N=299); mean age 46.9 years	1. Individualized tailoring: questionnaire about the individual's situation used to recommend resources<br>2. 3 modules focusing on components of knowledge and cognitive and behavioral skills including coping with emotions and difficulties	Waiting list	Caregiver strain; positive caregiving (Positive Aspects of Caregiving); depression (CES-D[a]); anxiety (State-Trait Anxiety Inventory); satisfaction survey	Improved stress (F=15.19, P<.001), self-efficacy (F=5.87, P=.02), intention to get support (F=9.76, P=.002), strain (F=4.90, P=.03), caregiver gain (F=5.35, P=.02), depression (F=6.83, P=.009), and anxiety (F=4.78, P=.03). No change in employing coping strategies. Positive correlation between time spent viewing the program and outcome (r=.30, P=.001).
Blom, 2015, Netherlands [24]	RCT<br>6 months	Family caregivers of PWD[b] with score >4 on CES-D or a	1. Multimedia lessons on problem solving, relaxation, cognitive restricting, and assertiveness; guided by a psychologist	Email newsletters with practical information about caring	Depression (CES-D); anxiety (HADS-A); pressure (Self-Perceived Pressure for Informal Care Scale); problems	Significant reduction in symptoms of depression and anxiety in intervention group, with differences in effect size 0.29 and 0.34, respectively,

		score >3 on HADS-A[c] or a minimum of 6 on a 1-item burden scale (N=251); mean age 61.2 years	2. Homework	and dementia	from dementia symptoms (RMBPC[d]); competence (SSCQ[e]); mastery (Pearlin Mastery Scale)	compared with comparison group.
Fowler, 2016, US [25]	RCT<br>4 months	Primary informal caregivers of homebound PWD (N=28); mean age 60 years	1. Peer interaction<br>2. Interaction with a multiprofessional health care team via a question-and-answer forum<br>3. Educational modules on providing care, self-care, and dementia<br>4. Links to community resources<br>5. Online sleep hygiene program<br>6. Blog where caregivers can upload pictures and share personal information	Usual care	General self-efficacy scale; insomnia severity index; sleep actigraphy band data	No significant difference in insomnia severity scores, sleep quality (underpowered to detect this), or self-efficacy as a result of the intervention.
Hattink, 2015, Netherlands, UK [26]	RCT<br>2-4 months	Informal caregivers, volunteers, and formal caregivers of PWD dwelling in the community (N=142); mean age 52 years	1. Modules covering information about dementia, practical advice on caring, and looking after the caregiver<br>2. Interaction with a learning advisor who tailored the program for the caregiver<br>3. Links to other resources including a Facebook forum	Waiting list group with no intervention for 4 months	Knowledge (Alzheimer's Disease Knowledge Scale); Alzheimer disease survey; approach (Approaches to Dementia Questionnaire); interpersonal reactivity (Interpersonal Reactivity Index); quality of life; burden; competence (SSCQ); evaluation of user experience of the	Rated positively by users for usefulness and user friendliness; statistically significant effects for informal caregivers on attitudes toward dementia (η^2=.19), distress (η^2=.15), empathic concern (η^2=.46), and taking the perspective of the PWD (η^2=.32). No significant impact on knowledge, quality of life or burden.<br>Significant reduction in self-

					intervention	reported sense of competence (η^2=.11); 59/142 dropped out, mostly for unknown reasons.
Hicken, 2016, US [27]	RCT<br>4-6 months	Caregivers of veterans with dementia (N=231); mean age 70.2 years	1. Video vignettes about dementia<br>2. Video vignettes and written information about caregiving<br>3. Assessments of caregiver well-being with proactive contact from a social worker or individualized tailoring if particular area of need<br>4. Self-guided cognitive behavioral therapy-based techniques for mood management<br>5. Links to local services	Telephone support, written information, and a digital video disc of the content	Burden (ZBI[f]); grief (Marwit-Meuser Caregiver Grief Inventory); mood (9-item Patient Health Questionnaire); family conflict and hardship; desire to institutionalize	Significant improvement in hardship (β=–.253, t_{104}=–2.66, *P*=.009) and grief (β=–.324, t_{48}=–2.46, *P*=.02) for the internet group compared with controls. All other outcome measures had no significant difference in change scores between cohorts.
Kajiyama, 2013, US [28]	RCT<br>3 months	Informal caregivers of PWD aged ≥21 years, caring for at least 8 hours per week (N=150); mean age 56 years	1. Education on dementia and planning for the future<br>2. Self-care advice including stress management techniques, relaxation, and healthy habits<br>3. Advice on communication skills and managing difficult behaviors	Website and written documents containing information on dementia only	Stress (a); problems with dementia symptoms (RMBPC); depression (CES-D); quality of life	31% dropped out.<br>Caregivers in the intervention group experienced significantly greater improvement in perceived stress (t_{45}=3.18, *P*=.003).<br>No significant change in any other outcome measures.
Nuñez-Naveira, 2016, Spain, Poland, Denmark [29]	RCT<br>3 months	Primary informal caregivers of PWD with a burden due to their caring	1. Chats and forums for peer discussion<br>2. Video, audio, and written content on instructions for caring tasks and information about dementia	Usual care	Depression (CES-D); burden (ZBI); usability questionnaires; competence (Caregiver Competence Scale); satisfaction (Revised	21% of caregivers dropped out. Statistically significant decrease in depressive symptomatology in the experimental group. No significant change in other

		(N=77)	3. Medication reminders<br>4. Calendar for caregivers to schedule events<br>5. Facility to record instructional videos for the care receiver<br>6. Questionnaire for individual customization<br>7. Signposting to other websites and resources		Caregiving Satisfaction Scale)	outcomes.
Van Mierlo, 2015, Netherlands [30]	RCT<br>12 months	Informal caregivers of PWD living at home (N=73); mean age 63.0 years	1. Tool to individually tailor information given on the basis of needs<br>2. Information about local and national care and support services	No access to the tool (DEM-DISC)	Needs (Camberwell Assessment of Needs for the Elderly); quality of life (Quality of life in Alzheimer's Disease and EuroQuol EQ5D+c); neuropsychiatric symptoms (Neuropsychiatric Inventory Questionnaire); competence (SSCQ); ease of use (Usefulness, Satisfaction and Ease of Use Questionnaire)	At 12 months: significantly more total care needs (*P*=.004) and total unmet needs (*P*=.02) in the experimental group compared with the control group; significantly higher sense of competence in the experimental group compared with control. No other significant differences.

Table 1. Characteristics, components, outcomes, and key findings of randomized controlled trials (RCTs).

[a]CES-D: Center for Epidemiologic Studies Depression Scale.

[b]PWD: person with dementia.

[c]HADS-A: Hospital Anxiety and Depression Scale.

[d]RMBPC: Revised Memory and Behavior Problems Checklist.

[e]SSCQ: Short Sense of Competence Questionnaire.

[f]ZBI: Zarit Burden Interview.
