# Supplementary material for: Internet-Based Interventions Aimed at Supporting Family Caregivers of People With Dementia: Systematic Review
Source: J Med Internet Res. 2018 Jun 12;20(6):e216. doi: 10.2196/jmir.9548 (PMC6019848; doi:10.2196/jmir.9548)
Supplement: Multimedia Appendix 3 [file jmir_v20i6e216_app3.pdf]

First author, year, country, reference	Study design, duration	Population	Core Components	Outcome measures	Key findings
Davis, 2015, US [31]	Pre-post study 2 weeks	Female, working caregivers of PWD[a] (N=5)	1. Peer support where caregivers upload videos of themselves and view others describing challenges of caring and ways to overcome these	Burden (ZBI[b]); Gain Through Group Involvement Scale	Burden was lessened by the information (data not given). Issues of security were raised.
Glueckauf, 2004, US [32]	Pre-post study 16 weeks	People providing care for an individual with dementia for at least 6 hours per week (N=21); mean age 64 years	1. 6 live Web-based interactive small-group classes addressing emotional aspects of caring led by a facilitator; participants could choose to join via website (n=15) or telephone (n=6)<br>2. Audiovisual presentations on dementia<br>3. Message board and chat room for peer interaction<br>4. Electronic information on dementia, caregiving techniques, and research developments<br>5. Links to other resources<br>6. Assignments	Self-efficacy (CSES[c]); Stress-related Growth Scale; caregiver appraisal (CAI[d])	Significant pre- to postintervention increases on all 3 CSES subscales (*F* range 6.07-6.91, *P*=.02) and decreases in subjective emotion burden subscale of the CAI (*F*=8.16, *P*=.01). No significant change in positive dimensions of the caregiving experience or stress.
Griffiths, 2018, US [33]	Pre-post study 6 weeks	People caring for a veteran with dementia (N=64); mean age 63.3 years	1. Daily internet-delivered video modules<br>2. Weekly group videoconferences run by a facilitator	Burden (ZBI); depression (CES-D[e]); caregiving mastery (Pearlin Mastery Scale); problem with dementia symptoms	Significant decrease in depressive symptoms (effect size 0.37), number of behavioral and psychological symptoms of dementia that occurred daily or

			3. Assignments	(Revised Memory and Behavior Problems Checklist)	more often (effect size 0.42), average behavioral and psychological symptoms of dementia frequency (effect size 0.32), and caregiver self-reported competence (effect size 0.61).<br>Caregivers' upset or distress with relation to behavioral and psychological symptoms of dementia increased after the program but not significantly.<br>A face-to-face program can feasibly be adapted to be delivered online.
Kajiyama, 2018, US [34]	Pre-post study 4 weeks	Hispanic or Latino people who are primary family caregivers of someone with dementia (N=25); mean age 57.4 years	1. Psychoeducation based on cognitive behavioral therapy delivered via telenovela episodes	Stress (Perceived Stress Scale); depression (CES-D), knowledge (Knowledge Survey)	Significant decrease in stress (*P*=.045), depression (*P*=.045), and knowledge (*P*=.04).
Kwok, 2014, Hong Kong [35]	Pre-post study 9 weeks	Primary caregiver for a PWD, who did not have depression (N=36); mean age not given	1. Information on basic caring skills, stress reduction, grief handling, and healthy lifestyle maintenance<br>2. Individualized online counselling based on cognitive behavioral therapy provided by messaging interaction with a professional	Neuropsychiatric symptoms (Neuropsychiatric Inventory Questionnaire); self-efficacy (Revised Scale for Caregiving Self-Efficacy)	Significant reduction in caregiver distress (Z=2.51, P<.05, r=.49) and severity of behavioral and psychological symptoms of dementia (Z=3.02, P<.01, r=.59). No significant difference in self-efficacy.
Lorig,	Pre-post study	Caregiver of a	1. Internet-based peer	Health (National Health	Significant reductions in burden

2012, US [36]	6-week intervention, follow-up at 3 months	veteran (or veteran themselves) with impaired cognition; caregiving at least 10 hours a week and have stress levels 4/10 or greater (N=60); mean age 57 years	discussion between 20-30 caregivers via discussion boards<br>2. Moderation by trained peer or professional facilitators<br>3. Interactive and didactic educational material released weekly covering self-care, decision making, and caring<br>4. Problem behavior diaries and exercise logs<br>5. Private communication between participants<br>6. List of resources including weblinks and documents	Interview Survey); illness intrusiveness (Illness Intrusiveness Index); visual numeric scales for pain, shortness of breath, stress, sleep, and fatigue; burden (ZBI); strain (Caregiver Strain Index); depression (8-item Patient Health Questionnaire); self-report sick days, effect of caregiving on work, living arrangements and the relationship between caregiver and recipient; self-report health-related behaviors and health care utilization	(effect size 0.220), depression (effect size 0.274), pain (effect size 0.321), and stress (effect size 0.696). Some self-reported health behaviors and feelings of self-efficacy also improved. There were no significant changes in health care utilization; 45% of participants logged in on all 6 weeks.
Van der Roest, 2010, Hong Kong [37]	Controlled pre-post study 2 months	Informal caregivers of PWD living at home with needs in areas addressed by the tool (DEM-DISC) (N=28); mean age 60.2 years	1. Tool to individually tailor information given on the basis of needs<br>2. Information about local and national care and support services<br>3. Information about dementia diagnosis and support on practical problems	Needs (Camberwell Assessment of Needs for the Elderly); use of services; competence (Short Sense of Competence Questionnaire); mastery (Pearlin Mastery Scale); quality of life (Quality of life in Alzheimer’s Disease); knowledge about care, welfare services and methods to	Informal caregivers reported more met needs (d=1.44), fewer unmet needs (d=−0.80), and higher sense of competence (d=0.93) after DEM-DISC use compared with controls. No significant differences were found for other outcome measures; informal caregivers were neutral in their opinions of the value of DEM-DISC.

				collect information; ease of use (Usefulness, Satisfaction and Ease of Use Questionnaire)	

Table 2. Characteristics, components, outcomes, and key findings of quasi-experimental (pre-post) studies.

[a]PWD: person with dementia.

[b]ZBI: Zarit Burden Interview.

[c]CSES: Caregiver Self-Efficacy Scale.

[d]CAI: Caregiver Self-Efficacy Scale.

[e]CES-D: Center for Epidemiologic Studies Depression Scale.
