# Supplementary material for: Internet-Based Interventions Aimed at Supporting Family Caregivers of People With Dementia: Systematic Review
Source: J Med Internet Res. 2018 Jun 12;20(6):e216. doi: 10.2196/jmir.9548 (PMC6019848; doi:10.2196/jmir.9548)
Supplement: Multimedia Appendix 4 [file jmir_v20i6e216_app4.pdf]

First author, year, country, reference	Study design, duration	Population	Core components	Outcome measures	Key findings
Kovaleva, 2017, US [38]	Semistructured interviews and online survey<br>7 weeks	People caring for a veteran with dementia (N=42); mean age 66 years	1. Daily internet-delivered video modules<br>2. Weekly group videoconferences run by a facilitator and composed of lectures and discussions<br>3. Assignments	Qualitative data	Most caregivers were positive about the intervention. The online format allowed them to form relationships and was convenient. Nonindividualized information was frustrating; there was insufficient information on early or later stages. The videos were valued but did not show enough ethnic diversity.
Marziali, 2005, Canada [39]	Interviews and observed support groups<br>6 months	Family caregivers of people with neurodegenerative disease (N=34); mean age 67.8 years	1. Virtual support groups led by a facilitator initially then by a group member<br>2. Disease-specific information<br>3. Question-and-answer forum<br>4. Videoconferencing link for one-to-one interaction<br>5. Private email access	Video observation of group sessions; qualitative interviews with participants	Positive responses from most participants to the intervention, including reduced isolation, good bonding with group members, improved psychological well-being, and usefulness to obtain information and support.
Marziali, 2006, Canada [40]	Interviews and observed support groups<br>6 months	Family caregivers of people with neurodegenerative disease (N=34); mean age not given	1. Virtual support groups led by a facilitator initially then by a group member<br>2. Disease-specific information<br>3. Question-and-answer forum<br>4. Videoconferencing link for one-to-one interaction<br>5. Private email access	Video observation of group sessions; qualitative interviews with participants	Most caregivers felt it was easy to use; most felt positively about using the internet to participate in a support group and found this convenient. The most frequently used aspect of the intervention was the disease-specific information.
Span, 2015, Netherlands [41]	Qualitative methods<br>5 months	Informal caregivers of a person with dementia (N=12),	1. Group chat function<br>2. “Deciding together” function for step-by-step decision making	Data derived from structured interviews, observations, and	Some usability problems, especially for older users; requested notification of new activities; all

		persons with dementia (N=4), and case managers (N=3); mean age of caregivers 54.3 years	3. Section for recording of individual opinions on 8 dementia-related topics	information that participants logged in the DecideGuide	liked the easy way of communicating within the network; some felt they did not need such a decision-making tool; no decisions were made with the tool, and instead it led to more face-to-face interactions where decisions took place.

Table 3. Characteristics, components, outcomes, and key findings of qualitative studies.
