# Supplementary material for: Internet-Based Interventions Aimed at Supporting Family Caregivers of People With Dementia: Systematic Review
Source: J Med Internet Res. 2018 Jun 12;20(6):e216. doi: 10.2196/jmir.9548 (PMC6019848; doi:10.2196/jmir.9548)
Supplement: Multimedia Appendix 5 [file jmir_v20i6e216_app5.pdf]

First author, year, country, reference	Study design, duration	Population	Core components	Outcome measures	Key findings
Austrom, 2015, US [42]	Mixed-methods study with pre-post intervention study and qualitative focus groups<br>6 months	Informal caregivers of people with dementia (N=4); mean age 56.2 years	1. Education via videoconference<br>2. Small-group peer support<br>3. Educational modules	Depression (PHQ-9[a]); anxiety (GAD-7[b]); health (SF-36[c]); caregiver burden; self-efficacy (RSCS[d]); postintervention focus groups	Improved depression (3.3), anxiety (1.5), and SF-36 physical score (2.7); worsened burden (–1.0); little change in self-efficacy (0.5) or SF-36 mental health score (0.3).<br>Focus groups appreciated ease of access from home and useful professionals, and valued peer support.
Boots, 2017, Netherlands [43]	Mixed-methods study with RCT[e] and qualitative interviews<br>8 weeks	Informal caregivers of people with dementia (N=49); mean age 69.6 years	1. Face-to-face introduction session with a psychiatric nurse or psychologist<br>2. Self-guided modules: video clips of family caregivers, text information<br>3. Assignments requiring self-reflection and a 5-step change plan<br>4. Forum for peer interaction<br>5. Email interaction with a coach	Process evaluation using quantitative questionnaires and qualitative interviews	The program was rated as a useful addition for family caregivers. They appreciated the tailored content. The discussion forum was appreciated least.
Boots, 2016, Netherlands [44]	Mixed-methods study with think-aloud usability testing with caregivers and experts, evaluation and a pre-post study<br>8 weeks	Pre-post study: spousal caregivers of people with mild cognitive impairment or mild dementia (N=10)	1. Face-to-face introduction session with a psychiatric nurse or psychologist<br>2. Self-guided modules: video clips of family caregivers, text information<br>3. Assignments requiring self-reflection and a 5-step change plan	Self-efficacy (CSES[f]); Likert evaluation scales	Postintervention, participants had significantly higher scores on CSES care management subscale (*P*=.03) and CSES service use (*P*=.01), with small effect sizes (0.14 and 0.41, respectively).<br>Caregivers appreciated the convenience of use from home, clarity, module structure, content,

			4. Forum for peer interaction<br>5. Email interaction with a coach		privacy, tailored assignments, and guidance by the coach. Most time was spent completing assignments (mean 79 minutes), least time on the forum (mean 7.5 minutes).
Brennan, 1995, US [45]	Mixed-methods study with an RCT and focus groups<br>12 months	Primary informal caregiver of someone with Alzheimer disease; able to read and write in English (N=102); median age 64 years	1. Peer interaction via a bulletin board and private mail<br>2. Online question-and-answer service with a nurse<br>3. Information on Alzheimer disease, its treatment, and caring<br>4. Decision support tool<br>Control: 1 face-to-face information session on caregiving and Alzheimer disease	Decision confidence scale; self-report decision-making skill; social support (Instrumental and Expressive Social Support Scale); impact of caregiving scale; depression (CES-D[g]); community and medical service contacts; dementia rating (Clinical Dementia Rating); focus groups	Improvement in decision-making confidence ($F_{1,93}$=9.73, P<.01).<br>No significant change in social isolation, decision-making skill, or health status.<br>Forum used more than other areas.<br>Focus groups: beneficial to communicate with peers and professionals at all times of day. Frustrations with speed of technology and recalling personal emails.
Chiu, 2009, Canada [46]	Mixed methods: pre-post study and in-depth qualitative interviews<br>6 months	Adults caring for a family member with dementia in the community who spoke, read, and wrote Chinese (N=35)	1. Online information handbook covering dementia information, caregiving strategies, and a list of community resources<br>2. Email communication between client and an occupational therapist or social worker	Burden (BSFC[h]); dementia symptoms (RMBPC[i]); depression (CES-D); health (Self Rated Health index); social support (MSPSS[j]); positive aspects (Positive Aspects of Caregiving); functional assessment (Multidimensional Functional Assessment Questionnaire); competence (Caregiver Competence Scale); in-depth interviews	Pre-post reduction in BSFC change score not statistically significant; younger caregivers significantly more likely to be frequent users, and older more likely to be nonusers.<br>In-depth interviews: some found the technology difficult, eg, logging on to the email account and typing and sending emails; some found the information handbook and contact with a professional supportive.

Cristancho-Lacroix, 2015, France [47]	Mixed-methods study: RCT and qualitative semistructured interviews<br>Data collection at 3 months and 6 months	Adult informal caregivers of people with Alzheimer disease providing care at least 4 hours per week with a score ≥12 on the PSS (n=49); mean age 62 years	1. 12 weekly multimedia sessions giving information on Alzheimer disease, caregiving, coping with difficult behaviors, and social and financial support<br>2. Forum for peer interaction, moderated by a clinical psychologist<br>3. Relaxation training<br>Control: unable to access the program, usual care from geriatrician	Stress (PSS[k]); self-efficacy (RSCS); dementia symptoms (RMBPC); burden (ZBI[l]); depression (Beck Depression Inventory); health (Nottingham Health Profile)	No significant difference in any of the scales other than knowledge of disease at 3 months (*d*=0.79, *P*=.008).<br>The most frequently visited section was the forum.<br>71% fully completed 10 of 12 sessions.<br>Qualitative data showed little acceptance of the program.<br>Caregivers wanted more interaction with a professional and peers, personalized support, and specific advice.
Dam, 2017, Netherlands [48]	Uncontrolled pilot study with repeated-measures design	Primary caregiver to someone with dementia in the community with access to the internet (N=25); mean age 55.9 years	1. Peer support and social interaction via network circles of family, friends, and significant others<br>2. Care book providing an overview of all contact and practical information relevant to the care process; ability to transfer care task to other members of their network<br>3. Links and signposting to relevant information on topics of dementia and caregiving	Perceived support (MSPSS); received support (12-item Social Support List); loneliness (Loneliness Scale); sense competence (Short Sense of Competence Questionnaire)	Mean evaluation 7.1/10 on Likert scale indicating acceptable feasibility.<br>Rate of low active users was high. However, overall reports were positive about the content and number of functions available.<br>Showed acceptable levels of satisfaction.<br>High active users showed less decline after 16 weeks of perceived family support (*P* not significant) than low users (mean difference –3.18, SE 1.12, t_{16}2.9, *P*=.01). High active users had lower levels of received support at 16 weeks (mean difference –2.50, SE 0.81, t_{22}=3.1, *P*=.03) compared with low active users, but this was not significant.
Hattink,	Mixed-methods	Informal caregivers of	1. Peer support	Usability information:	Large variability between

2016, Netherlands [49]	assessment of usability: observation, online surveys and semistructured interviews	PWD[m], people with dementia, and memory clinic health professionals<br>Observation: N=10, online survey: N=287, semistructured interviews: N=18	2. Interaction with a health care professional<br>3. Information on dementia and caring<br>4. Links to community and national events and resources	ease of use, attractiveness, appreciation of the content; errors and time on tasks	participants in time and number of errors made in completing tasks. Most caregivers and patients who used the portal rated it positively with regard to usability and considered it a useful addition to existing care.
Jajor, 2016, Poland [50]	Quantitative usability evaluation<br>2 months	Informal caregivers of people with Alzheimer disease (N=18); mean age 57 years	1. Chats and forums for peer discussion<br>2. Video, audio, and written content on instructions for caring tasks and information about dementia<br>3. Medication reminders<br>4. Calendar for caregivers to schedule events<br>5. Facility to record instructional videos for the care receiver<br>6. Questionnaire for individual customization<br>7. Signposting to other resources	Usability of the technology	Contrasting colors made the app easy to use. Instructions on how to use the intervention were valuable. Finding specific information was sometimes difficult for caregivers.
Lewis, 2010, US [51]	Mixed-methods evaluation of participant experiences: quantitative scales and qualitative free text<br>4 months	Informal caregivers of PWD (N=63); mean age 55 years	Four modules covering the following:<br>1. Information on the effects of dementia on thinking<br>2. Information on the practical aspects of caring<br>3. Advice on “taking charge and letting go”<br>4. How to manage daily care and difficult behavior	Experiences of using the program using Likert scales and qualitative free text	Quantitative: 94% gained confidence in caring; 96% gained new ideas and strategies for caring; 100% felt more confident communicating with the PWD; 92% enjoyed learning on the internet as opposed classes.<br>Qualitative: caregivers liked the information and caregiving strategies, videos, convenience, and presentation.<br>They did not like technical

					difficulties, repetition of information, the length, or lack of interaction with others.
Marziali, 2006, Canada [52]	Mixed methods: RCT and qualitative analysis<br>6 months	Family caregivers of people with neurodegenerative disease (N=66); mean age 67.8 years	1. Virtual support groups led by a facilitator initially then by a group member<br>2. Disease-specific information<br>3. Question-and-answer forum<br>4. Videoconferencing link for one-to-one interaction<br>5. Private email access<br>Control: no intervention	Health (HSQ-12[n]); depression (CES-D); a measure of activities of daily living performed on behalf of the care recipient; symptoms of dementia (RMBPC); social support (MSPSS)	Statistically significant effect of the intervention on stress change score ($F_{1,37}$=9.68, *P*=.004).<br>Qualitative data: most felt positively about meeting online.
Marziali, 2011, Canada [53]	Mixed methods: pre-post study and qualitative interviews<br>6 months	Caregivers of PWD who live with the care recipient (N=91); mean age 65.5 years	1. Online information about dementia and self-care<br>2. Private email with peers<br>Videoconference group also had the following:<br>3. Online videoconferencing supportive group sessions run by a therapist for 10 weeks and then by a group member for a further 12 weeks<br>Chat group also had the following:<br>3. Library of dementia educational videos<br>4. Text-based chat forum	Health (HSQ-12); dementia (CES-D); functional autonomy (Functional Autonomy Measurement System); self-report service use	Both groups showed significant improvement in self-efficacy (*F*=4.3, *P*<.04). Compared with the chat group, the video group showed significantly greater improvement in mental health status (*F*=5.69, *P*<.02) and lower distress scores associated with managing the care recipient's deterioration in cognitive function (*F*=5.65, *P*<.02). On average, participants in the chat group used the chat forum once in 6 months. Video group participants attended, on average, 7 of the facilitated sessions and 5 of the self-help sessions.<br>Qualitative: varied opinions of participants on the chat function; video group felt that this was a helpful way to get emotional support.

McKechnie, 2014, UK [54]	Mixed methods: pre-post study and qualitative interviews<br>12 weeks	Informal adult caregivers of a PWD on their first visit to the forum (N=61); mean age 56 years	1. Online forum	Anxiety (GAD-7); depression (PHQ-9); relationship quality (Scale for the Quality of the Current Relationship in Caregiving)	29% did not visit the forum, 76% visited <12 times. Statistically significant improvement in quality of relationship (d=–0.78). No significant change in anxiety or depression. Significant correlation between time on forum and reduction in PHQ-9 scores (ρ=.356, P=.03).<br>Qualitative themes: reduced isolation and loneliness; normalization of experiences; provides information, sometimes not available elsewhere; anonymity important; flexible to use.
O'Connor, 2014, US [55]	Mixed methods: pre-post study and qualitative comments<br>8 weeks	Informal caregivers of a PWD (N=10); mean age 60.9 years	1. Virtual support groups providing peer support and education on managing difficult behaviors and unhelpful thoughts, communication, planning for the future, and pleasant events	Loneliness scale; Geriatric Depression Scale; burden (ZBI); stress (PSS); qualitative comments on usability	30% dropped out. Mean changes were in the expected direction of improvement for all outcome measures, but power was insufficient for these differences to reach statistical significance.<br>Qualitative data: found useful factual information; felt generally supported; useful to access from home.
Pagán-Ortiz, 2014, US [56]	Mixed methods: controlled pre-post study and qualitative focus groups<br>1 month	Spanish-speaking caregivers of PWD (N=72); no mean age given	1. Information about dementia and managing difficult behaviors<br>2. Advice on self-care<br>3. Peer support forum<br>4. Ask an expert resource<br>5. Links to national and international resource	Mastery (Pearlin Mastery Scale); social network (Lubben Social Network Scale); burden (ZBI); depression (CES-D); focus group responses	No significant change in any outcomes in comparison with the control group.<br>Qualitative data: the website was valuable and convenient; the Ask an Expert section was not often used but was helpful for those who used it; most

					participants preferred to read information; recommendations for improvement included information on spirituality, local resources and medication therapies, and including dementia specialist physicians in the "Ask an Expert" section.
Pot, 2015, Netherlands [57]	Quantitative evaluation of the intervention<br>6 months	Family caregivers of PWD with score >4 on CES-D or score >3 on HADS-A° or >6 on a burden scale (N=149); mean age 61.5 years	1. Multimedia lessons on problem solving, relaxation, cognitive restricting, and assertiveness; guided by a psychologist<br>2. Homework<br>3. Information on dementia and caregiving	Depression (CES-D); anxiety (HADS-A); tracking of lessons opened; reasons for not completing entire intervention; quantitative evaluation of each lesson	45.6% completed all lessons. Among completers, there were more spouses (73.5% vs 48.1%, *P*=.002), more living in same household (76.5% vs 49.4%, *P*=.001), and higher age of caregivers (63.8 vs 59.7 years, *P*=.04).<br>Completers had a mean score of 3.84/5 for usefulness of the lessons and 4.16/5 for feedback from the coach.
Rentz, 2010, US [58]	Quantitative evaluation of the website	Family caregiver of a PWD (N=121)	1. Selected educational materials<br>2. Journal<br>3. Health and well-being surveys<br>4. Online peer forums<br>5. A social worker who communicated with caregivers by email, journal entries, and occasional phone conversations	Survey data on experience of use	The vast majority of caregivers responded positively to the intervention agreeing they had improved understanding of dementia, felt supported, and felt better prepared to cope with caregiving problems.
Schaller, 2015, Germany [59]	Mixed methods: quantitative and qualitative evaluation of the	Adult informal caregivers of a PWD (N=31); mean age 58 years	1. Personalized portal<br>2. Electronic diaries to record caring, course of disease, and medication	Likert scale; semistructured interviews	Caregivers perceived good support from the intervention; the provision of individualized information on dementia treatment, local support

	intervention		3. Decision-making support<br>4. Online communication with a professional with telephone follow-up as necessary<br>5. Personalized information on dementia and caring		services, and strategies for preventing caregiver burden were most useful; concerns were raised around privacy and data security; improvements included a search function and peer interaction.
Schaller, 2016, Germany [60]	Mixed methods: pre-post study and qualitative semistructured interviews<br>12 weeks	Adult informal caregivers of a PWD (N=25); mean age 58 years	1. Personalized portal<br>2. Electronic diaries to record caring, course of disease, and medication<br>3. Decision-making support<br>4. Online communication with a professional with telephone follow-up as necessary<br>5. Personalized information on dementia and caring	Empowerment (Carers' Needs Assessment for Dementia); decision making (Preparation for Decision Making Scale); quality of life (EuroQoL-5-dimensional 5-level questionnaire); burden (BSFC); perceived benefits using a Likert scale; semistructured interviews	The most perceived empowerment was achieved via information acquisition; 65% would use the intervention; no pre-post change in quality of life, burden.<br>Qualitative: caregivers felt major benefits were time saved, 24-hour access, facilitated communication with professionals, ease of use, and having an overview about caregiving activities; concerns were raised around data privacy, nonusage, insufficient internet competence, and how to assure up-to-date information.
Weitzman, 2008, US [61]	Mixed methods: pre-post study and qualitative written evaluation after 2-4 hours' use of the website	Latino adults for pre-post study (N=31); Latino caregivers of a PWD for evaluation (N=12); no mean age given	1. Instructions on providing personal care<br>2. Financial and long-term care planning advice<br>3. Links to local caregiver resources<br>4. Information on dementia<br>5. Advice on dealing with difficult behavior	Self-reported knowledge; attitudes toward the website	Self-reported knowledge improved; caregivers rated the content and design positively; problems with print size and length of content in some sections.

Table 4. Characteristics, components, outcomes, and key findings of mixed-methods and other methods studies.

[a]PHQ-9: 9-item Patient Health Questionnaire.

[b]GAD-7: 7-item Generalized Anxiety Disorder Questionnaire.

[c]SF-36: 36-Item Short Form Health Survey.

[d]RSCS: Revised Scale for Caregiving Self-Efficacy.

[e]RCT: randomized controlled trial.

[f]CSES: Caregiver Self-Efficacy Scale.

[g]CES-D: Center for Epidemiologic Studies Depression Scale.

[h]BSFC: Burden Scale for Family Caregivers.

[i]RMBPC: Revised Memory and Behavior Problems Checklist.

[j]MSPSS: Multidimensional Scale of Perceived Social Support.

[k]PSS: Perceived Stress Scale.

[l]ZBI: Zarit Burden Interview.

[m]PWD: person with dementia.

[n]HSQ-12: 12-item Health Status Questionnaire.

[o]HADS-A: Hospital Anxiety and Depression Scale.
